# Falls in Geriatric Populations and Hydrotherapy as an Intervention: A Brief Review

**DOI:** 10.3390/geriatrics3040071

**Published:** 2018-10-18

**Authors:** Alana J. Turner, Harish Chander, Adam C. Knight

**Affiliations:** Neuromechanics Laboratory, Mississippi State University, Mississippi State, MS 39762, USA; hchander@colled.msstate.edu (H.C.); aknight@colled.msstate.edu (A.C.K.)

**Keywords:** geriatric falls, hydrotherapy, aging, postural control, balance training

## Abstract

Falls and fall-related injuries are a serious health concern in geriatric populations, especially with age-related deficits in postural control and during postural control challenging dual-task situations. Balance training has been reported to be beneficial in reducing falls. However, some of these exercises have their inherent physical challenges that prevent the elderly population from performing them effectively. Other concomitant age-related illness in the elderly pose further challenges in performing these exercises. Hence, the topic of finding alternative types of balance training that are effective and are performed in a safer environment is constantly researched. One such alternative is hydrotherapy that focuses on balance and postural perturbation-based exercises in water-based environments such as aquatic swimming pools or in dedicated hydrotherapy pools. Hydrotherapy for geriatric populations has been reported to be beneficial in improving balance, motor and cognitive tasks with improved motivation and positive attitude towards exercises. Additionally, hydrotherapy also has properties of buoyancy, resistance and temperature, which benefit biomechanical and physiological wellness and offers a safe environment to perform balance training. Hydrotherapy balance training need to be scaled and prescribed according to individual needs and can serve as an effective training and rehabilitation protocol in reducing falls in geriatric population.

## 1. Introduction

By the year 2020, one out of five people will be 70 years of age or older [1]. The natural aging process is accompanied by changes in cognitive and sensory domains that lead to balance impairments, resulting in the recurrence of falls [2]. Further, the possibility of recurrent falls doubles after the first fall which will qualify such an elderly individual as a “high-risk” patient [3]. Falls for an older population is classified as a geriatric syndrome ultimately leading to higher mortality, morbidity, and medical costs for the United States [1]. Falls are known to be multifaceted; however, the disruption of postural control is one factor that can lead to falls and fall-related injuries. Postural control is the ability to maintain a state of equilibrium and spatial orientation in an upright stance with the hindrance of gravitational forces. Postural control is essential to maintaining daily activities such as walking and balance [4]. Disruption or ineffective functioning of the sensory and motor components of the postural control system leads to an increased likelihood of falls in the elderly. Finding effective ways to prevent falls in the geriatric population may reduce disability arising from falls and fall-related injuries and subsequently increase life expectancy of these individuals.

## 2. Postural Control in the Aging Population

For the human body, over half its mass resides in the torso and upper extremity regions [5]. This creates an unbalanced environment for most individuals, especially the geriatric population. Constantly maintaining balance has become an imposing task for most elderly individuals. About 33% of elderly adults fall each year, resulting in moderate to severe injuries [3]. In 2017, The Centers for Disease Control and Prevention reported that 2.8 million senior adults are treated in emergency room hospitals for fall injuries each year [1]. Therefore, falls are now considered a leading public health problem among the elderly population [6]. Horak and colleagues refer to postural control as a multifaceted skill maintained by multiple sensorimotor systems [7]. Horak determined the factors responsible for postural control include higher center processing, control of dynamics, spatial orientation, biomechanical factors, sensory and movement strategies [7]. These factors of postural control tend to decline over time as a natural function of aging and subsequently this decline results in an increased prevalence of falls for geriatric population [7]. The natural course of aging changes the neuromuscular and sensorimotor systems by negatively affecting balance performance in dynamic and static postural control and subsequently leading to fall-related accidents [8]. Ultimately, a combination of factors results in a disturbance of postural control which leads to a sequence of events; an individual’s postural control system being disrupted or getting exposed to a perturbation, leading to an inability to maintain upright equilibrium, losing balance, unable to recover from the perturbation and subsequently falling, which leads to fall-related injuries that could be of musculoskeletal or neuromuscular in origin.

Accidents from environment-related surroundings was the most frequently noted, accounting for 30–50% in most literature [9]. In particular, during accidents, the amount of stability an individual maintains is dependent on the movement of Center of Mass (COM) and Base of Support (BOS) parameters. The geometrical shape involving the COM and BOS creates an inverted pendulum, or a cone. McCollum and colleagues refer to this as an individual’s stability cone [10]. The stability cone and COM movement determine the type of strategy adopted by an elderly population [7]. Three discrete strategies discussed in pervious literature are ankle, hip, and stepping strategies [7]. Elderly people tend to embrace the hip or stepping strategy to maintain balance because of multisensory deficits [9]. Shupert and colleagues elaborated that the preferred hip strategy maintains COM by adjusting the body along the inverted pendulum with counter motion at the hip and ankle [11]. Moreover, the elderly experience multiple impairments due to the regression of their sensorimotor systems and these impairments can be a result of muscle weakness, sensory loss, and cognitive dysfunction [10]. These deficiencies correlate directly with immobility, negatively impacting daily functions such as climbing stairs, walking, and overall independent living [10].

Proprioception is an important aspect of postural control and balance, especially in geriatric populations. Goble and colleagues suggested decline in proprioceptive function in these population, making them more prone to the loss of balance and subsequent falls [12]. Given the rising percentage of individuals over the age of sixty, these deficits have encouraged increased interest regarding the proprioceptive improvements of the elderly population, and the role of proprioceptive feedback in elderly movement. One of the main problems older individuals are faced with is controlling the time duration of muscle contractions to assist with efficient multi-joint movement in postural control [12]. The underlying problem of age-related decreases in proprioceptive feedback makes exercises and coordinated movement more difficult for this population, thereby contributing to physical inactivity.

## 3. Dual-Task Paradigm Leading to Falls

Balance deficiencies are among the most commonly known risk factors contributing to falls, with individuals being up to 5.4 times more likely to fall if they exhibit poor balance and cognitive function [3,7]. Moreover, the elderly population commonly experience falls while performing two activities simultaneously; in other words, dual tasking involves the performance of one task (postural control task) while also completing a second task (cognitive or motor task) [3]. Previous literature reported that dual tasking has different levels of difficulty and therefore places differing levels of demand on the individual’s cognitive processing systems, affecting fine and gross motor tasks [3]. Much research has focused on the emphasis of postural control and dual task activities, known as the dual task paradigm. This is because most falls occur while older adults execute two activities simultaneously, one of which is maintaining postural control [3]. Ultimately, a trade-off between postural control and cognitive/motor task is reached, which can negatively impact either one or both of the tasks, depending upon the complexity of the postural control and cognitive/motor demands. For example, response times and performance in a cognitive task have been reported to decline as postural control task difficulty increases [7]. Because postural control systems occupy the same ascending and descending pathways as cognitive functioning, the performance of the postural task will decline with a second cognitive task [7]. Some individuals in the geriatric population have a limited amount of cognitive processing due to neurological deficiencies; hence, the available cognitive processing network to control posture may be limited [7]. Moreover, reoccurrence of falls due to dual task activities led researchers to form studies involving attentional demands and dual tasking to incorporate a combination of cognitive and motor tasks [2]. Furthermore, the ability to maintain postural stability is reduced when performing two or more tasks concurrently [13]. Previous research suggests that elderly adults who perform poorly under dual-task conditions are more likely to experience falls [13]. According to Bergamin and colleagues, different dual task conditions affect postural stability. For example, a verbal task demonstrated the largest deficits of postural balance. Other variables, aside from age, also affect center of pressure sway-velocity measurements. For instance, an increase in center of pressure sway-velocity was observed while counting backwards, whereas a decrease was reported during memory-related tasks [14]. Additional research has shown that with a simultaneous walking and talking task, participants would take a longer time to complete their gait task, alter their gait pattern, or stop walking altogether [13]. Also, Kim and colleagues discuss the need for incorporating rhythm-motor tasks at different levels with cognitive-motor tasks. The combination of cognitive-motor dual tasks with rhythmic cueing can add external auditory sources as a compensatory strategy to maintain gait stability within the dual task paradigm [15]. These findings solidify the notion that balance performance is influenced while simultaneously performing a cognitive and/or motor task [13]. Conclusively, a higher incidence of falls results from insufficient cognitive processing to control posture while occupied with a second cognitive or motor tasks [7].

## 4. Balance Training for Fall Prevention

With the main biomechanical restriction faced by geriatric population being maintaining balance, Shumway-Cook and Brauer suggest that balance control and training helps maintain activities of daily living such as walking, placing a book on a high shelf, and cleaning, in a geriatric population [7,16]. The degenerative changes of aging result in lowered inputs of the somatosensory, vestibular, and visual processes, and slower conduction speeds of the central nervous system, which affects balance performance [7,16]. Many therapeutic exercise interventions have been used in an attempt to prevent the risk of falling in the elderly population. Static and dynamic balance training can improve postural control and decrease the risk of falls for geriatric population [17]. Sherrington et al. recommends balance training when the population’s main goal is to reduce the risk and rate of falls [18]. Balance training with regards to fall prevention targets improvements of postural control by challenging an individual’s body alignment of his/her center of gravity in relation to their base of support [19]. Furthermore, dose-response relationships are also very important to consider when introducing a balance training intervention. Multiple balance training protocols revealed that a training period of 6–12 weeks, a frequency of three sessions per week, a total number of 36–40 training sessions, a duration of a single training session of 31–45 min, and a total duration of 91–120 min of balance training per week is most effective to improve balance [8].

## 5. Hydrotherapy as an Intervention for Fall Prevention

While most of the land-based balance training exercises are effective, they have their own inherent complications and physical challenges that prevent a successful training for geriatric population, demanding a need for alternative forms of balance training for elderly populations in a safe and efficient manner. The few risk factors that can lead to falling include a previous history of falls, age, lower extremity weakness, environmental settings, proprioception deficits, and fear of falling. Many healthcare professionals recommend safe physical activity to patients to improve proprioception and to help maintain a better quality of life [20]. In addition, different types of therapeutic physical activity interventions have been researched to improve postural control [19]. Fall prevention exercise programs have been created for the elderly population and have been successfully implemented as well; however, some exercises on land can be difficult due to joint pain and lack of muscular endurance [21].

Hydrotherapy is the use of exercises while the body is emerged in water [20]. Hydrotherapy has been widely applied to therapeutic fields for injury rehabilitation and training interventions to improve muscular strength, balance, and cardiovascular fitness [20]. The properties of water, which include buoyancy, resistance, and temperature, combined with physical exercises, can help relieve many physiological issues of natural ageing and promote physical activity [22]. The aquatic environment is considered safe and efficient for the rehabilitation of elderly people [22], and water provides a supportive, low risk exercise environment that may reduce the likelihood of acute injury and fear of falling while improving participation and adherence [23]. The weight-relieving property of water immersion allows for smoother movements with less pain, which may also be a result from the warmth of the water. Furthermore, previous literature has shown that hydrotherapy can help decrease the many risk factors of falling for the elderly population [10]. Weightless physical activity, via hydrotherapy, has been shown to improve motor-related tasks and cognitive processes while in a safe setting for an elderly population [24]. Some studies have reported that hydrotherapy can improve musculoskeletal impairments within six weeks of programming compared to land-based exercises [24]. Furthermore, many researchers have noted that the buoyancy of an aquatic environment decreases the amount of gravity placed on the body, creating a weightlessness setting [25]. This type of weightlessness environmental training can be easier for elderly patients to exercise and improve dynamic and static balance [24]. Also, underwater exercises create a safe environment for training balance while preventing falls and decreasing the fear of falling [25]. 

Devereux et al. reported that benefits of hydrotherapy include improved motor control, strength, mobility, balance, and task-oriented movements [26]. One of the benefits observed after a four, six, and eight-week aquatic exercise program was improved balance. Hale et al. reported that a 6-week program resulted in significant improvement in postural sway in women 70 years of age [27]. Moreover, previous studies have reported that a minimum of 90 min a week for 6 weeks of hydrotherapy training can improve balance skills for the elderly and help to reduce the prevalence of falls and fall-related injuries [8]. The exercise prescription of each session should include the exercises below from the table. Each session includes a 5-min warm-up and stretch period, 20-min balance and strengthening/conditioning exercises with pool buoys and noodles, and a 5-min cool down where the instructor emphasizes some of the important aspects of the session, relating to body and skill awareness. The groups progress through each level throughout the six weeks, placing emphasis on Level 5 exercises the last two weeks. Hydrotherapy provides the geriatric population with a sense of confidence and a better quality of life, especially for individuals in a community-based dwelling [27]. For this type of population, instilling confidence can be just as crucial as improving physical health [27]. Moreover, a group aquatic exercise program creates a motivational environment. Motivation challenges people to improve their well-being and overall improved the psychosocial aspects of a test group, according to Reference [27]. Moreover, a more positive attitude from participants after the exercise program was observed [27]. Even though there is limited literature in which the effectiveness of hydrotherapy in geriatric populations in a dual-task paradigm is tested, it has been reported to provide greater improvements in balance tests, postural control, and weight-bearing exercises while performing single task activities [18].

**Figure 1:**
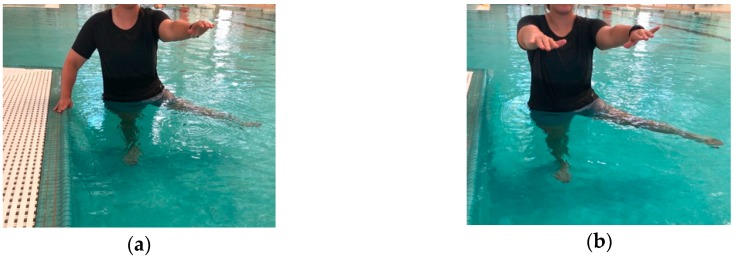
(**a**) Level 1 Exercise: Standing with support hand support from the wall, wide-base foot stance, lifting one foot from the pool base at a time. (**b**) Level 2 Exercise: Same as Level 1, but no external support.

**Figure 2:**
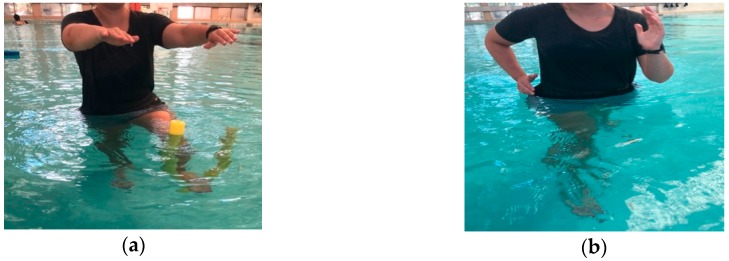
(**a**) Level 3 Exercise: Standing in unilateral stance while the opposite leg flexes and extends the knee with “noodle” under the foot. (**b**) Level 4 Exercise: Cutting in different directions (e.g., anteriorly/posteriorly and medial/lateral), at differing speeds and in deeper water.

**Figure 3:**
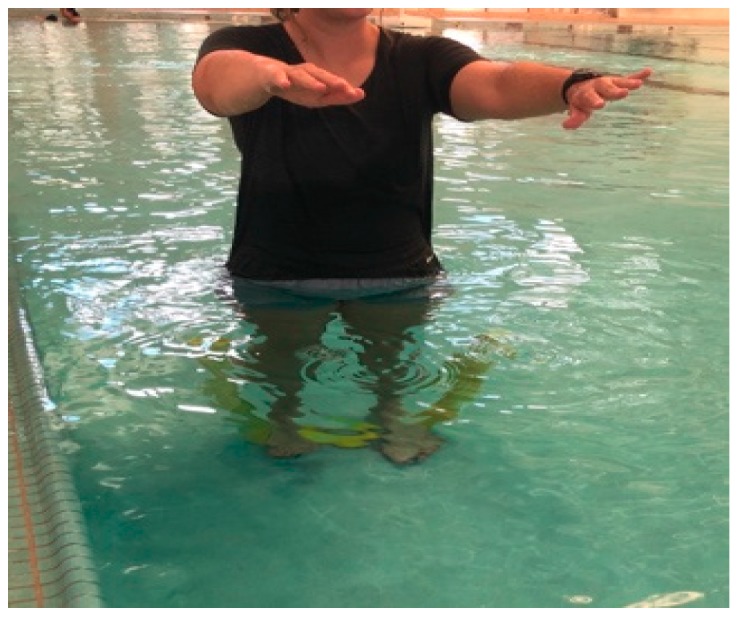
Level 5 Exercise: Similar to level 4 but while standing on noodle, shifting base of support, while opening/closing eyes, adding cognitive tasks.

Finally, this paper attempts to offer suggestions for hydrotherapy exercise prescription for elderly individuals based on adaptations of research contributions from Oddson et al. in hydrotherapy, which provides five levels of training protocols that gradually progress in intensity and complexity of the exercises performed [28,29]. An instruction type exercise protocol for these five levels, based on adaptations from Oddson et al. [28,29] are provided in figures (Figure 1, Figure 2 and Figure 3) and table (Table 1). As such, these hydrotherapy prescriptions can be used as suggested protocols in an elderly population in an attempt to reduce falls and fall-related injuries.

## 6. Clinical Applications

As individuals age, postural stability decreases due to many factors which makes rehabilitation and exercise difficult for the geriatric population. As a take-away for healthcare professionals and for clinical application purposes, hydrotherapy is a safe, effective form of rehabilitation and exercise for the geriatric population. Hydrotherapy programs could be prescribed more by physicians to help encourage a safe environment for exercise to improve balance and coordination for this population to decrease the number of falls and instill confidence in their patients. Moreover, Gobbo and colleagues reported that most exercise programs for the elderly population, such as Tai Chi and chair training, provided inconclusive evidence for improving static and dynamic balance. Thus, new clinical approaches, such as hydrotherapy, could help improve balance performance to prevent falls [30]. 

## 7. Conclusions

In geriatric population, postural control decreases due to many factors, which predisposes these population to an increased risk for falls. These factors responsible for reduced postural control can be both intrinsic (internal) to the human body that can be attributed to the physiological ageing process, and extrinsic (external) factors due to environmental constraints that present physical challenges to maintain an erect posture without falling. Hydrotherapy offers a successful training and rehabilitation method to help improve postural control, confidence, mobility and quality of life in elderly population, especially in a group setting. The training can easily be scaled at different levels for different levels of complexity of the exercise program that best fits the individual and can be gradually progressed with exercises of increasing complexity. Thus, hydrotherapy offers a successful alternative method of balance training in preventing falls and fall-related injuries in geriatric population.

## Figures and Tables

**Table 1:** An example of various levels of hydrotherapy-based balance training exercises for geriatric populations based on adaptations from Oddson et al. [28,29].

Level	Hydrotherapy Exercises
**Level 1 & 2:** Sit to stand exercises with external support (Level 1 exercises are performed with external support while Level 2 exercises are performed without external support)	Standing next to pool wall, wide base with feet, one hand support on pool wall. Repeat with shoulder-width stance and support.
	Wide-base foot stance with hand support on wall, shift body weight in different directions as much as possible.
	Standing with pool wall hand support, and twisting trunk right and left as much as possible.
	Standing with support hand on the wall, wide-base foot stance, lift one foot from the pool base at a time. Repeat while in narrow stance.
	Standing with support, wide-base foot stance, and introduce leaning forward into the water.
	Shifting the base of support or closing eyes will increase the intensity of the exercises.
**Level 3:** Standing exercises including bi-lateral stance with external support	Standing in unilateral stance while the opposite leg flexes and extends the knee with “noodle” under the foot.
	Same as above, while throwing or catching a ball.
	Standing with wide/narrow foot base, holding a “noodle” with both hands and trying to drive it into the water while staying upright.
	Sitting on a “noodle” and staying upright with chest out of the water.
	Standing with both feet on the “noodle” and staying upright, with or without a cognitive task.
	Standing with both feet on a “noodle”, holding a “noodle” with both hands and trying to drive it into the water while staying upright.
	Cutting in different directions (e.g., anteriorly/posteriorly and medial/lateral), at differing speeds and in deeper water.
**Level 4:** Standing exercises including uni-lateral stance, walking with no external support	Cutting directions as quickly as the subject can withstand.
	Walking on a “noodle” with and without differing cognitive tasks.
	Adding neck movements to challenge the vestibular system.
	Similar to levels 3 and 4 but with added water turbulence and in different water depths.
**Level 5:** Pushing exercises, reaction responses	Standing, wide base stance, while instructor pushes the participant in different directions, with and without knowledge.
	Similar to level 4 but while standing on noodle, shifting base of support, while opening/closing eyes, adding cognitive tasks.
	Walking and being pushed by instructor/classmates with and without additional cognitive tasks.
